# Supplementary material for: UNC-43/CaMKII-triggered anterograde signals recruit GABAARs to mediate inhibitory synaptic transmission and plasticity at C. elegans NMJs
Source: Nat Commun. 2023 Mar 15;14:1436. doi: 10.1038/s41467-023-37137-0 (PMC10015018; doi:10.1038/s41467-023-37137-0)
Supplement: Supplementary file 1 — Supplementary Information [file 41467_2023_37137_MOESM1_ESM.pdf]

Supplementary Information

UNC-43/CaMKII-triggered anterograde signals recruit $GABA_A$Rs to mediate inhibitory synaptic transmission and plasticity at *C. elegans* NMJs

Hao, *et al*.

Supplementary Figures

Supplementary Figure 1

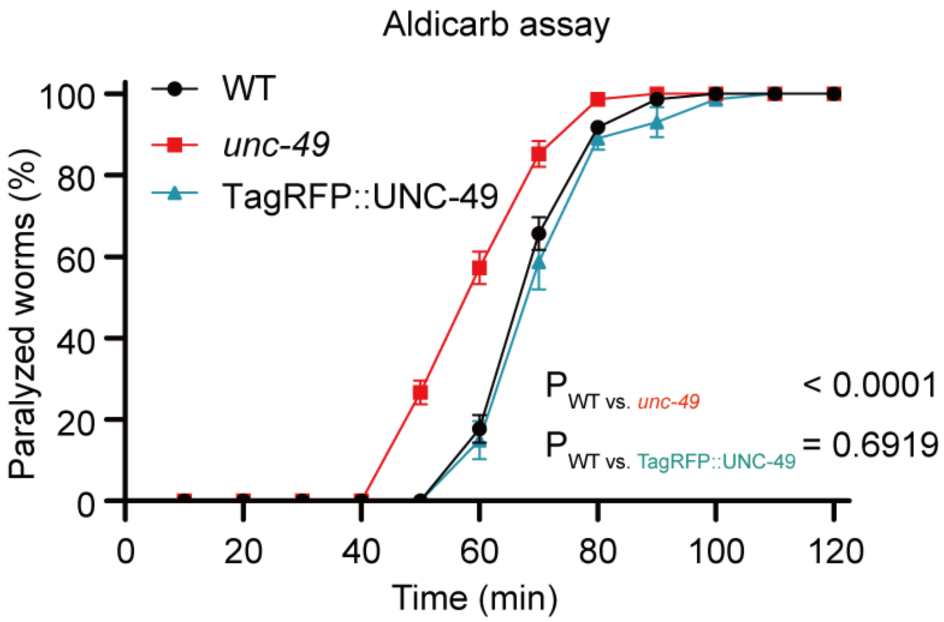

Supplementary Figure 1. The aldicarb sensitivity is not altered by tagRFP-UNC-49 fusion. Aldicarb assay is used to detect synaptic transmission of NMJs in wild type (black), *unc-49* (e407) mutants (red), and transgenic animals with *xj1024* allele expressing tagRFP-UNC-49 fusion (blue). For each of the group, n=3 biologically independent samples; each sample contains ≥25 animals. Data are presented as mean values +/- SEM. Two-way ANOVA comparing all of the time points. *** p<0.001, n.s. not significant. Source data are provided as a Source Data file.

Supplementary Figure 2

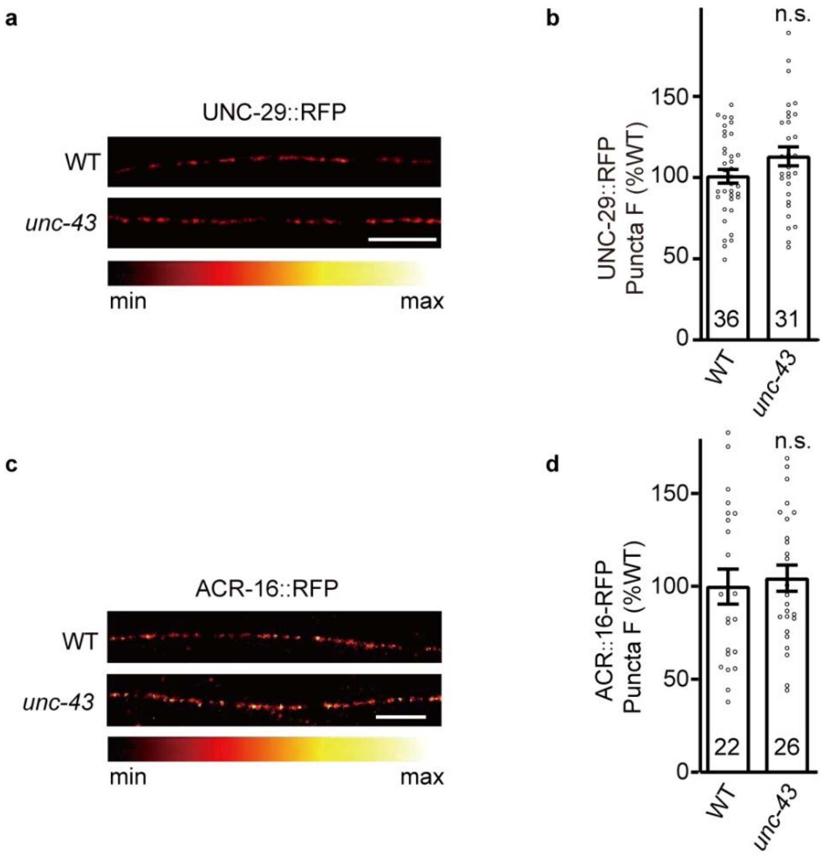

Supplementary Figure 2. The postsynaptic AChRs abundance is not altered in the *unc-43* mutants. a, b UNC-29-RFP puncta fluorescence is comparable in wild type and *unc-43* mutants. Representative images (**a**, scale bar 10 μm, Pseudo-color: Red Hot) and mean puncta intensities +/- SEM (**b**, Ns represent the number of animals tested) are shown. Two-tailed and unpaired Student's t-test. n.s. not significant. **c, d** The puncta fluorescence intensity marked by the acetylcholine receptor ACR-16-RFP is unaltered in the *unc-43* mutant. Representative images (**c**, scale bar 10 μm, Pseudo-color: Red Hot) and mean puncta intensities +/- SEM (**d**, Ns represent the number of animals tested) are shown. Two-tailed and unpaired Student's t-test. n.s. not significant. For **b**, **d**, source data are provided as a Source Data file.

Supplementary Figure 3

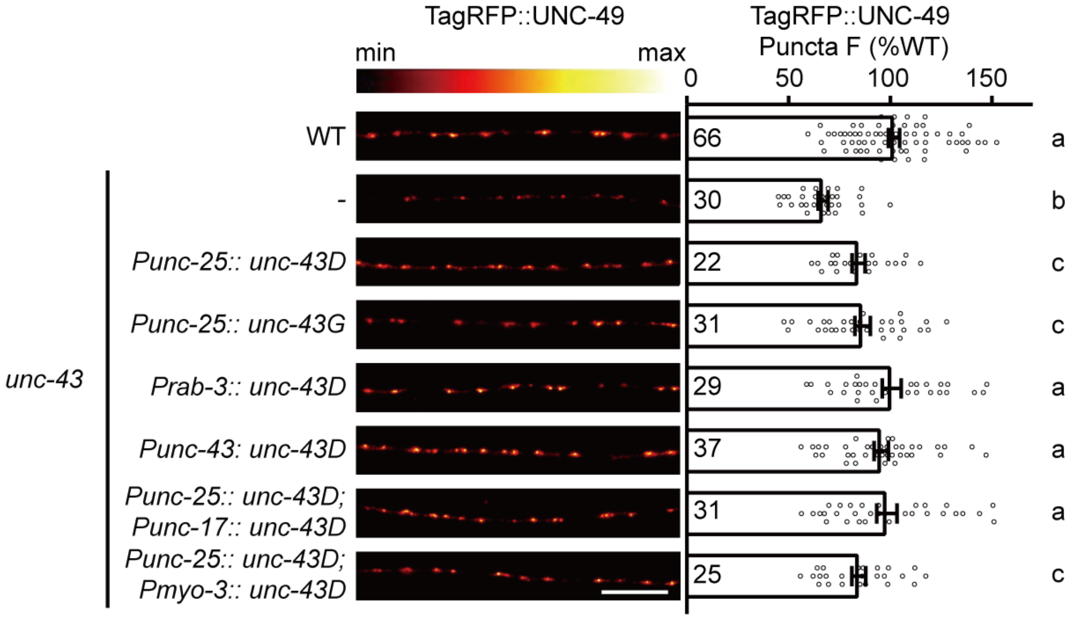

Supplementary Figure 3. Rescue of GABA$_A$Rs recruitment defect in *unc-43* mutants. The decreased TagRFP-UNC-49 puncta fluorescence intensity was rescued by transgenic expression of UNC-43 D or G isoform in GABAergic motor neuron (under *unc-25* promoter), endogenous expressing cells (under *unc-43* promoter), pan neuron (under *rab-3* promoter) both GABAergic motor neurons (under *unc-25* promoter) and cholinergic motor neurons (under *unc-17* promoter), and both in GABAergic motor neurons (under *unc-25* promoter) and body-wall muscles (under *myo-3* promoter). Representative images (left, scale bar 10 µm, Pseudo-color: Red Hot) and mean puncta intensities +/- SEM (right, Ns represent the number of animals tested) are shown. One-way ANOVA with two-stage linear step-up procedure of Benjamini, Krieger, and Yekutieli correction for multiple comparisons. Data corresponding to scatter plot labeled with different letters are significantly different ($p<0.05$). Source data are provided as a Source Data file.

Supplementary Figure 4

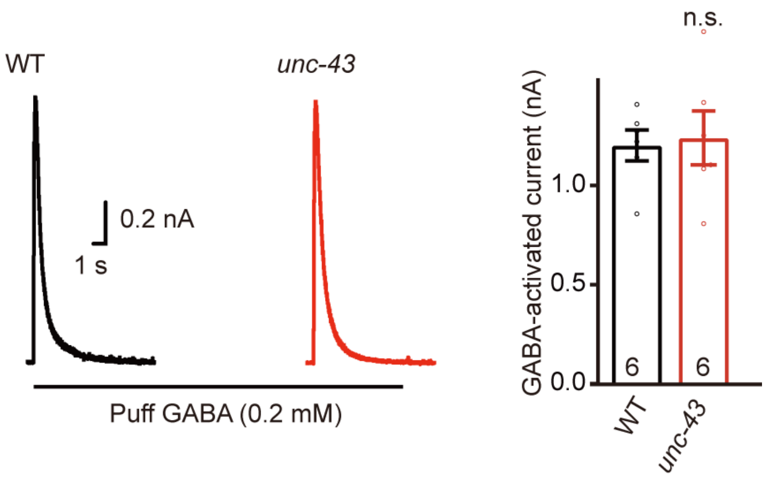

Supplementary Figure 4. The GABA-evoked currents in the *unc-43* mutants were comparable to that in wild-type animals (WT). Representative responses and mean current amplitudes +/- SEM are shown. Ns represent the number of animals tested. Two-tailed and unpaired Student's t-test. n.s. not significant. Source data are provided as a Source Data file.

Supplementary Figure 5

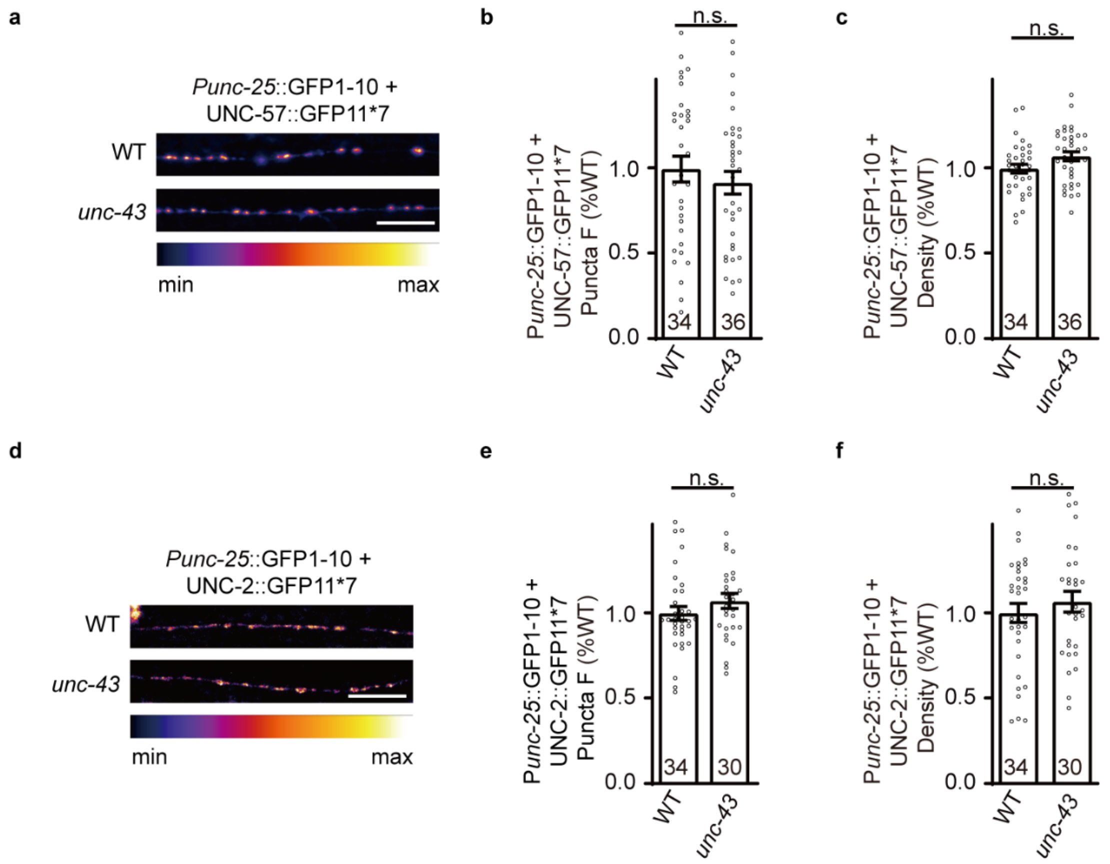

Supplementary Figure 5. GABAergic synapse structure is unaltered by deletion of *unc-43*. a-c The puncta fluorescence intensities and densities—marked by the GABAergic UNC-57::split GFP (under *unc-25* promoter)—are unaltered in the *unc-43* mutants. Representative images (**a**, scale bar 10 μm, Pseudo-color: Fire), mean puncta intensities +/- SEM (**b**, Ns represent the number of animals tested) and mean puncta densities +/- SEM (**c**, Ns represent the number of animals tested) are shown. Two-tailed Mann Whitney test for **b** and Two-tailed and unpaired Student's t-test for **c**. n.s. not significant. **d-f** Split GFP complementary system to label the endogenous UNC-2 at the GABAergic synapses. The puncta fluorescence intensities and densities—marked by the GABAergic UNC-2::split GFP (under *unc-47* promoter)—are unaltered in the *unc-43* mutants. Representative images (**d**, scale bar 10 μm, Pseudo-

color: Fire), mean puncta intensities +/- SEM (**e**, Ns represent the number of animals tested) and mean puncta densities +/- SEM (**f**, Ns represent the number of animals tested) are shown. Two-tailed and unpaired Student's t-test for **e** and **f**. n.s. not significant. For **b**, **c**, **e**, **f**, source data are provided as a Source Data file.

Supplementary Figure 6

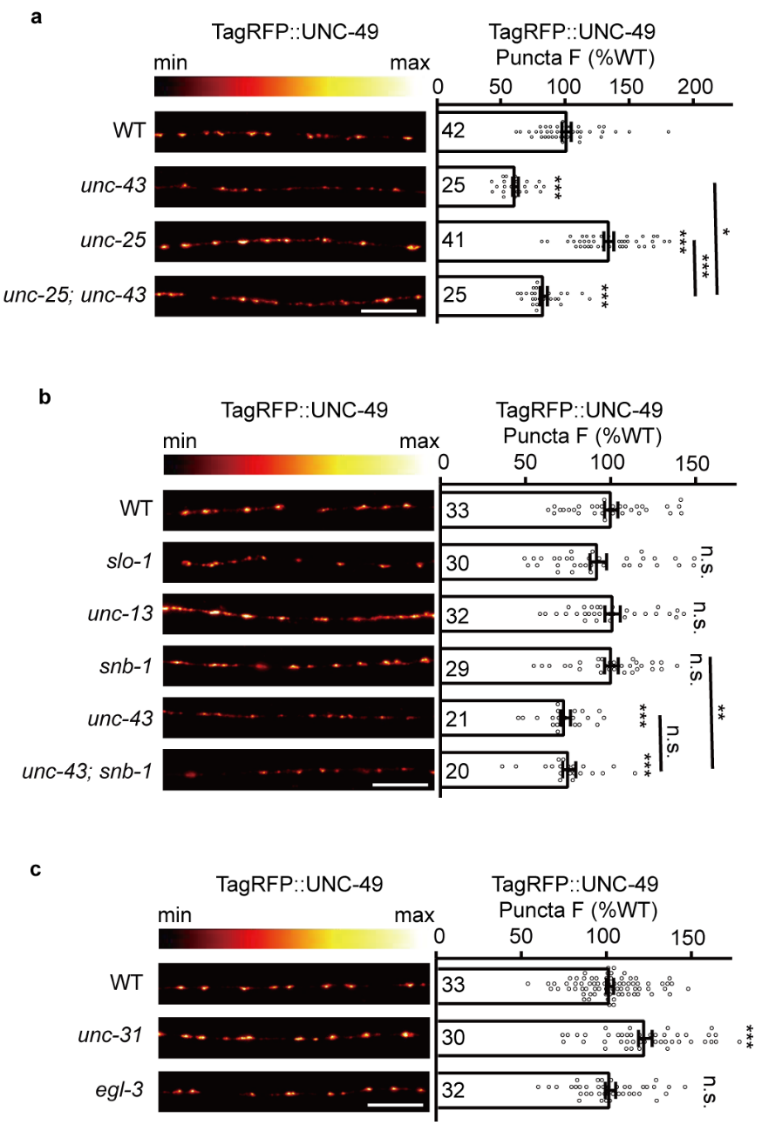

Supplementary Figure 6. Lack of synaptic vesicle or dense-core vesicle release does not cause a decrease in GABA$_A$R abundance at synapses. TagRFP-UNC-49 puncta fluorescence intensities in *unc-25*, *unc-25; unc-43* (**a**), *slo-1*, *unc-13*, *snb-1*, *unc-43; snb-1* (**b**), *unc-31* and *egl-3* (**c**) mutants. Representative images (left, scale bar 10 µm, Pseudo-color: Red Hot) and mean puncta intensities +/- SEM (right, Ns represent the number of animals tested) are shown. Kruskal-Wallis test with post-hoc Dunn's test for **a**, one-way ANOVA with two-stage linear step-up procedure of Benjamini, Krieger, and Yekutieli correction for multiple comparisons for **b** and one-

way ANOVA with post-hoc Bonferroni's multiple comparison test for **c**. * p<0.05, ** p<0.01, *** p<0.001, n.s. not significant. For **a-c**, source data are provided as a Source Data file.

Supplementary Figure 7

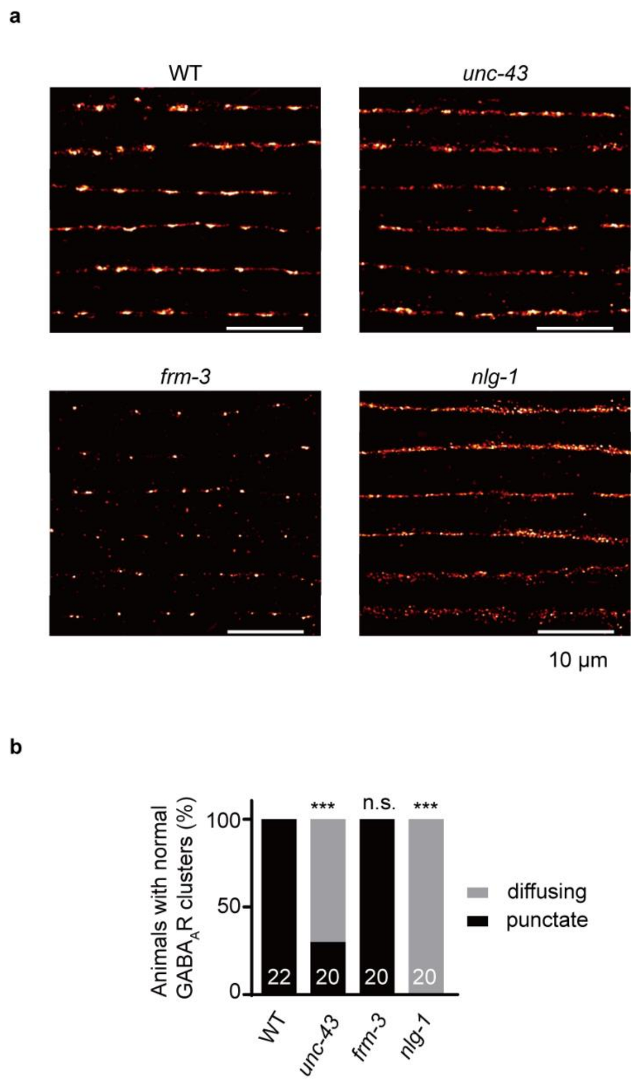

Supplementary Figure 7. $GABA_A$Rs clustering is impaired in the *unc-43* mutants. Quantification of $GABA_A$Rs clustering in the *unc-43*, *frm-3*, and *nlg-1* mutants by Super-resolution microscopy studies (Sora mode). Representative images (**a**, scale bar 10 µm, Pseudo-color: Red Hot) and the percentage of animals with normal $GABA_A$R clusters (**b**, Ns represent the number of animals tested) are shown. Chi-square tests. ***, p<0.001, n.s. not significant. Source data are provided as a Source Data file.

Supplementary Figure 8

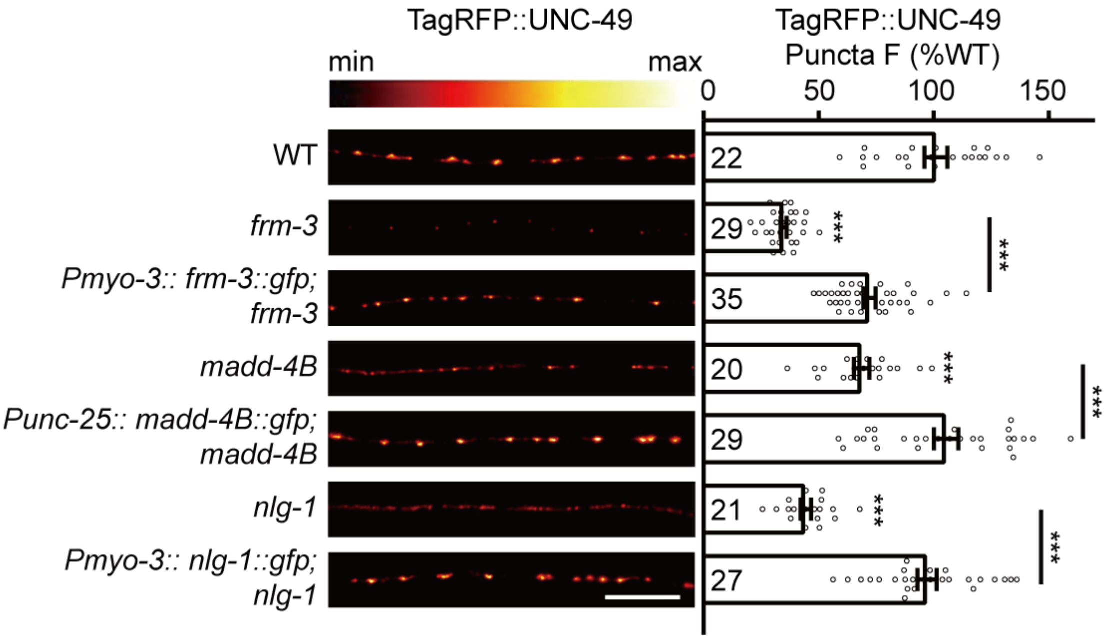

Supplementary Figure 8. Fusion proteins are able to rescue the GABA$_A$Rs recruitment defects in their corresponding mutants. TagRFP-UNC-49 fluorescence in dorsal nerve cords in wild type and mutants is shown. Representative images (left, scale bar 10 µm, Pseudo-color: Red Hot) and mean puncta intensities +/- SEM (right, Ns represent the number of animals tested) are shown. One-way ANOVA with two-stage linear step-up procedure of Benjamini, Krieger, and Yekutieli correction for multiple comparisons. ***, p<0.001. Source data are provided as a Source Data file.

Supplementary Figure 9

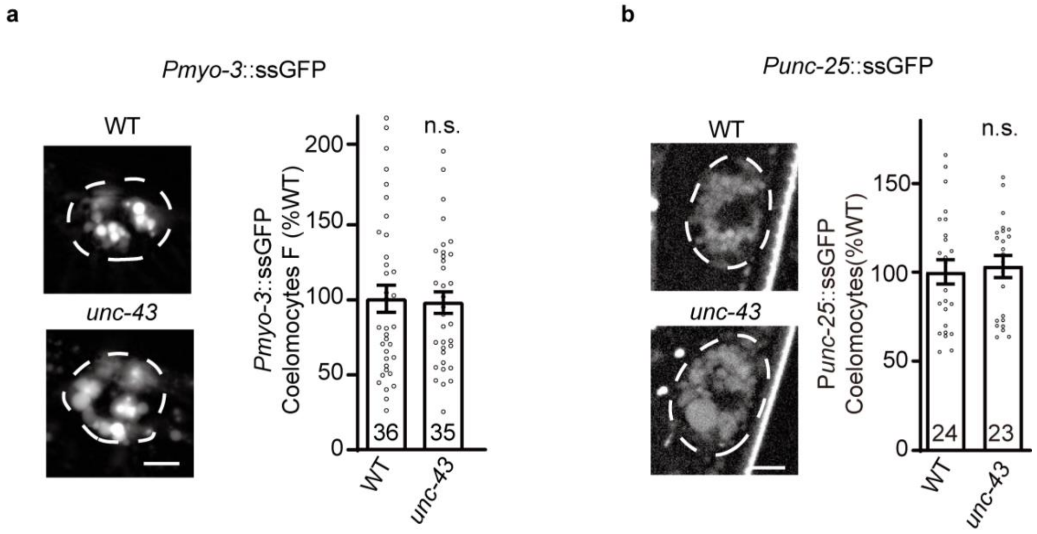

Supplementary Figure 9. The endocytosis of coelomocytes and general presynaptic protein secretion are not affected by *unc-43* mutation.

a The endocytosis of muscle cell-secreted GFP by coelomocytes was not affected in the *unc-43* mutants. **b** The constitutive secretion of GFP from GABAergic motor neuron terminals was not affected in the *unc-43* mutants. In **a** and **b**, Secretion of GFP was measured by analyzing GFP fluorescence intensities in the coelomocytes. The representative images (left, Scale bar 5 μm.) and the mean fluorescence intensities +/- SEM (right, Ns represent the number of animals tested) are shown (right panel). Two-tailed Mann Whitney test. n.s. not significant. For **a**, **b**, source data are provided as a Source Data file.

Supplementary Figure 10

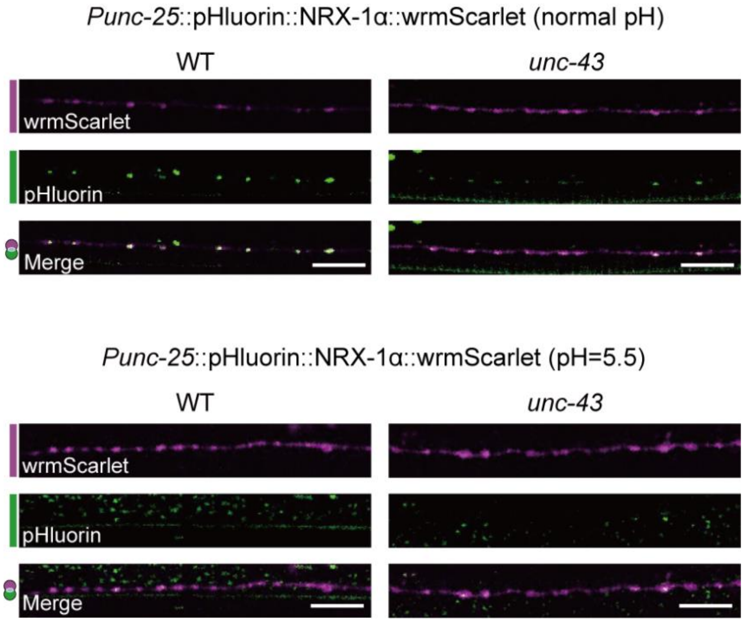

Supplementary Figure 10. pHluorin-NRX-1α puncta fluorescence was quenched by acidification of the extracellular environment. pHluorin and wrmScarlet dual-labeled NRX-1α was expressed in GABAergic neurons. pHluorin (green) and wrmScarlet (magenta) puncta fluorescence in the dorsal nerve cords were measured in animals with the normal (upper panel, PH > 6) or acidified (lower panel, PH=5.5) extracellular environment. Representative images (Scale bar 10 μm) are shown. Each experiment was repeated independently in more than 5 animals.

Supplementary Figure 11

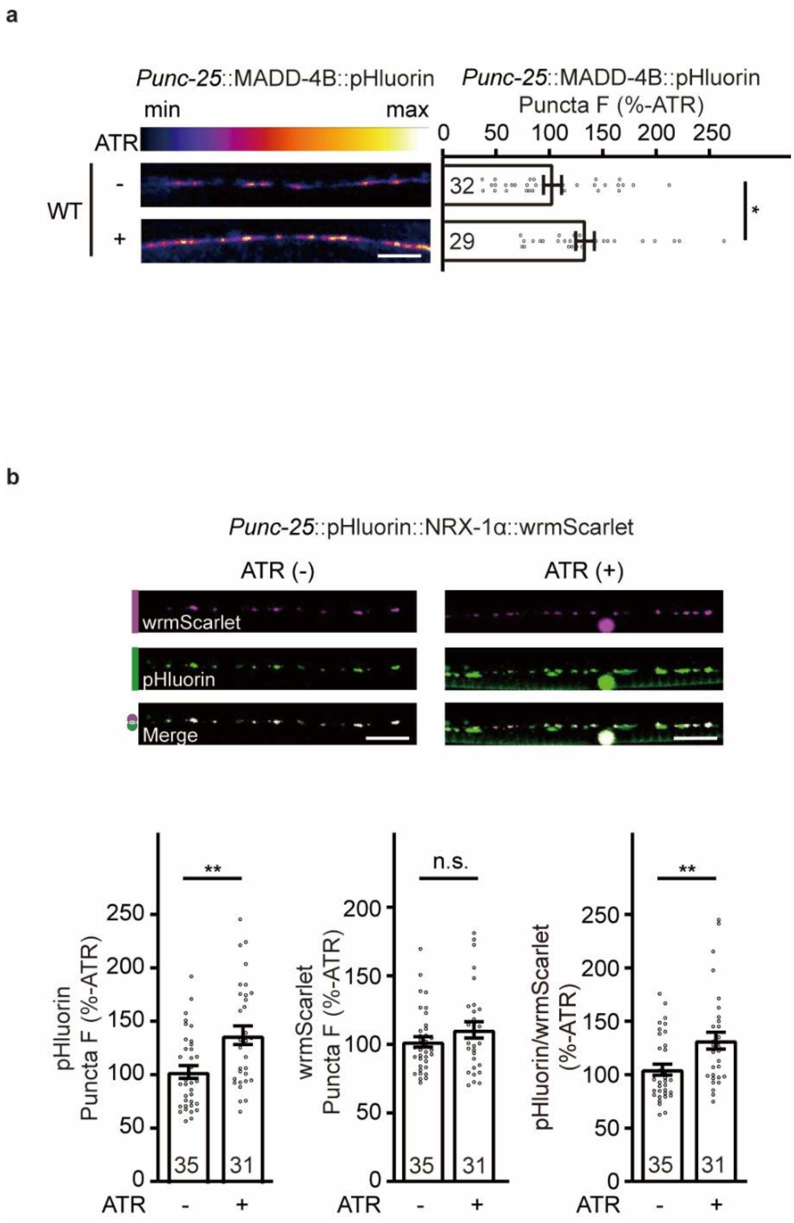

Supplementary Figure 11. The secretion of MADD-4B and surface delivery of NRX-1α were increased after GABAergic motor neuron excitation by ChIEF activation. a The pHluorin fusion tag was fused at the N-terminus of MADD-4B, and its fluorescent signal indicates the secreted MADD-4B that was retained at synaptic membranes. pHluorin-MADD-4B puncta fluorescence intensity in the dorsal nerve cords was increased in transgenic animals expressing a channelrhodopsin variant (ChIEF) in the GABAergic motor neurons (under *unc-25* promoter) after blue-light stimulation with all-trans retinal (ATR). Representative images (left, scale bar 10 μm, Pseudo-color: Red Hot) and mean puncta intensities +/- SEM (right, Ns represent the number of animals tested) are shown. Two-tailed Mann Whitney test. * $p<0.05$. **b** The surface localization of NRX-1α is increased in transgenic animals expressing ChIEF in the GABAergic motor neurons after blue-light stimulation with all-trans retinal (ATR). Representative images (upper, scale bar 10 μm), the averaged fluorescence intensity of pHluorin (lower, left), wrmScarlet (lower, middle), and the fluorescence intensity of pHluorin normalized to wrmScarlet (lower, right) are shown. Data are presented as mean values +/- SEM. Ns represent the number of animals tested. Two-tailed Mann Whitney test. ** $p<0.01$, n.s. not significant. For **a**, **b**, source data are provided as a Source Data file.
