# Supplementary material for: UNC-43/CaMKII-triggered anterograde signals recruit GABAARs to mediate inhibitory synaptic transmission and plasticity at C. elegans NMJs
Source: Nat Commun. 2023 Mar 15;14:1436. doi: 10.1038/s41467-023-37137-0 (PMC10015018; doi:10.1038/s41467-023-37137-0)
Supplement: Supplementary file 3 — Description of Additional Supplementary files [file 41467_2023_37137_MOESM3_ESM.docx]

**Description of Additional Supplementary Files**

**Supplementary Data 1:** List of *C. elegans* strains used in this study

**Supplementary Data 2:** List of DNA constructs generated and used in this study

**Supplementary Data 3:** List of oligonucleotides used in this study

**Supplementary Data 4:** List of Reagents, Software, and Algorithms used in this study
