# Supplementary material for: UNC-43/CaMKII-triggered anterograde signals recruit GABAARs to mediate inhibitory synaptic transmission and plasticity at C. elegans NMJs
Source: Nat Commun. 2023 Mar 15;14:1436. doi: 10.1038/s41467-023-37137-0 (PMC10015018; doi:10.1038/s41467-023-37137-0)
Supplement: Supplementary file 8 — Reporting Summary [file 41467_2023_37137_MOESM8_ESM.pdf]

nature portfolio

Corresponding author(s): Xia-Jing Tong

Last updated by author(s): Feb 10, 2023

Reporting Summary

Nature Portfolio wishes to improve the reproducibility of the work that we publish. This form provides structure for consistency and transparency in reporting. For further information on Nature Portfolio policies, see our Editorial Policies and the Editorial Policy Checklist.

Statistics

For all statistical analyses, confirm that the following items are present in the figure legend, table legend, main text, or Methods section.

n/a	Confirmed	
[ ]	[x]	The exact sample size (*n*) for each experimental group/condition, given as a discrete number and unit of measurement
[ ]	[x]	A statement on whether measurements were taken from distinct samples or whether the same sample was measured repeatedly
[ ]	[x]	The statistical test(s) used AND whether they are one- or two-sided<br>*Only common tests should be described solely by name; describe more complex techniques in the Methods section.*
[x]	[ ]	A description of all covariates tested
[ ]	[x]	A description of any assumptions or corrections, such as tests of normality and adjustment for multiple comparisons
[ ]	[x]	A full description of the statistical parameters including central tendency (e.g. means) or other basic estimates (e.g. regression coefficient) AND variation (e.g. standard deviation) or associated estimates of uncertainty (e.g. confidence intervals)
[ ]	[x]	For null hypothesis testing, the test statistic (e.g. *F*, *t*, *r*) with confidence intervals, effect sizes, degrees of freedom and *P* value noted<br>*Give P values as exact values whenever suitable.*
[x]	[ ]	For Bayesian analysis, information on the choice of priors and Markov chain Monte Carlo settings
[x]	[ ]	For hierarchical and complex designs, identification of the appropriate level for tests and full reporting of outcomes
[ ]	[x]	Estimates of effect sizes (e.g. Cohen's *d*, Pearson's *r*), indicating how they were calculated

Our web collection on statistics for biologists contains articles on many of the points above.

Software and code

Policy information about availability of computer code

Data collection	For fluorescense imaging, images were captured using an Olympus BX53 microscope or a Nikon spinning-disk Yokogawa CSU-W1 confocal microscope.
Data analysis	The following software was used:<br>ImageJ v1.48;<br>Igor pro 6.3;<br>GraphPad Prism 8;<br>MetaMorph v7.8.0

For manuscripts utilizing custom algorithms or software that are central to the research but not yet described in published literature, software must be made available to editors and reviewers. We strongly encourage code deposition in a community repository (e.g. GitHub). See the Nature Portfolio guidelines for submitting code & software for further information.

Data

Policy information about availability of data

All manuscripts must include a data availability statement. This statement should provide the following information, where applicable:

- Accession codes, unique identifiers, or web links for publicly available datasets
- A description of any restrictions on data availability
- For clinical datasets or third party data, please ensure that the statement adheres to our policy

The data supporting the findings of this study are included within the article and its Supplemental files. Reagents are available from the corresponding author upon reasonable request. The source data underlying the quantification of Figure 1c-d, 2d, 3b-c, 3f-g, 3i, 4a-d, 5a-c, 6b-c, 7c-e, 8c, 8e, 8g-h, 9a, 9c-d and Supplementary Figure 1, 2b, 2d, 3, 4, 5b-c, 5e-f, 6a-c, 7b, 8, 9a-b, 11a-b are provided as a Source Data file.

Human research participants

Policy information about studies involving human research participants and Sex and Gender in Research.

Reporting on sex and gender	N/A
Population characteristics	N/A
Recruitment	N/A
Ethics oversight	N/A

Note that full information on the approval of the study protocol must also be provided in the manuscript.

Field-specific reporting

Please select the one below that is the best fit for your research. If you are not sure, read the appropriate sections before making your selection.

☒ Life sciences ☐ Behavioural & social sciences ☐ Ecological, evolutionary & environmental sciences

For a reference copy of the document with all sections, see nature.com/documents/nr-reporting-summary-flat.pdf

Life sciences study design

All studies must disclose on these points even when the disclosure is negative.

Sample size	Sample sizes were based on previous experiments that used comparable methodology and were previously published by our laboratory (e.g. Xia-jing Tong 2015, Elife. Xia-jing Tong 2017, Neuron. Kang-ying Qian 2021, Elife. etc.). For each Figure, The sample size of animals were present in figures or figure legend.
Data exclusions	For imaging, electrophysiology assays, and aldicarb assay, the outliers were excluded by graphpad prism Identify outlier analysis followed with ROUT method (Q=10%). No other data were excluded.
Replication	At least three independent biological replicates were performed for each experiment. Replicate experiments yielded the same results.
Randomization	For each experiment, we randomly picked about 50 worms on the NGM plates which contained about 400 worms as experimental samples. And the worms in the same genetic background looks identical. So the animals chosen for imaging and electrophysiology were random.
Blinding	The aldicarb assay, double-blind replicates were performed for each genotype. For the analysis of fluorescence levels, colocalization and electrophysiology, all data were acquired and analyzed using the same settings, which does not require blinding. This method of analysis was previously published (Jihong Bai 2010, Cell. Zhitao Hu 2012, Science. Xia-jing Tong 2015, Elife. Xia-jing Tong 2017, Neuron. Kang-ying Qian 2021, Elife.).

Reporting for specific materials, systems and methods

We require information from authors about some types of materials, experimental systems and methods used in many studies. Here, indicate whether each material, system or method listed is relevant to your study. If you are not sure if a list item applies to your research, read the appropriate section before selecting a response.

Materials & experimental systems

n/a	Involved in the study
☒	☐ Antibodies
☒	☐ Eukaryotic cell lines
☒	☐ Palaeontology and archaeology
☐	☒ Animals and other organisms
☒	☐ Clinical data
☒	☐ Dual use research of concern

Methods

n/a	Involved in the study
☒	☐ ChIP-seq
☒	☐ Flow cytometry
☒	☐ MRI-based neuroimaging

Animals and other research organisms

Policy information about studies involving animals; ARRIVE guidelines recommended for reporting animal research, and Sex and Gender in Research

Laboratory animals	This study used C. elegans Bristol N2 as wildtype background, al the allele in this study can be found in Supplementary data 1. The well-fed young-adult hermaphrodites were used in all experiments except for coelomocyte imaging experiment in which the adult day-5 animals were used.
Wild animals	Nematode N2 which is commonly used in studies performed on C.elegans. No wild animals used in this study.
Reporting on sex	The hermaphrodite worms were used for all of the experiments in this study.
Field-collected samples	Study did not involve specimens collected from the field.
Ethics oversight	No ethical approval or guidance was needed.

Note that full information on the approval of the study protocol must also be provided in the manuscript.
